# Room temperature 90° phase-matching in zirconium and magnesium co-doped lithium niobate crystals

**DOI:** 10.1038/s41598-018-22205-z

**Published:** 2018-03-01

**Authors:** Tengfei Kong, Hongde Liu, Xinyu Ge, Da Qu, Shiguo Liu, Shaolin Chen, Ling Zhang, Yongfa Kong, Romano Rupp, Jingjun Xu

**Affiliations:** 1 School of Physics, Nankai University, Tianjin, 300071 China; 2 MOE Key Laboratory of Weak-Light Nonlinear Photonics and TEDA Institute of Applied Physics, Nankai University, Tianjin, 300457 China; 3 R&D Center, Taishan Sports Industry Group, Leling, 253600 China; 4 Collaborative Innovation Center of Chemical Science and Engineering (Tianjin), Tianjin, 300072 China; 5 Vienna University, Faculty of Physics, A-1090 Wien, Austria

## Abstract

Laser has been widely used in many aspects, by now it is difficult to get each frequency that we want, and frequency conversion is an effective way to obtain different frequency laser through a nonlinear optical crystal. MgO-doped LiNbO_3_ (Mg:LN) crystal has usually been used for second harmonic generation (SHG) through temperature-matching configuration with a stove, till now a room temperature 90° phase-matching is still lacking. Here we find that the SHG of Nd:YAG laser is achieved at 26.1 °C while the optical damage resistance is higher than 6.5 MW/cm^2^ in the ZrO_2_ and MgO co-doped LiNbO_3_ (Zr,Mg:LN) crystal. Moreover, the monotonic decrease of phase-matching temperature is firstly found with the increase of doping concentration. These unusual properties may be attributed to the formation of ${{\bf{Mg}}}_{{\bf{Li}}}^{{\boldsymbol{+}}}$  + ${{\bf{Zr}}}_{{\bf{Nb}}}^{{\boldsymbol{-}}}$ defect pairs. Our work suggests that Zr,Mg:LN crystal may be an attractive candidate for nonlinear optical applications.

## Introduction

Miniature, compact and high-power all-solid-state blue-green lasers are widely applied in ocean exploration, medical treatment, optical communication and laser display, etc^1–4^. As one of the most versatile optical crystals, lithium niobate (LiNbO_3_, LN) plays a key role in the second harmonic generation (SHG) of blue-green light from near-infrared lasers owing to its large nonlinearity and capability of temperature-tuned noncritical phase matching (90° phase-matching)^5–8^. Unfortunately, optical damage in LiNbO_3_, also known as photorefractive effect, induced by modest intensities of visible light, sharply decreases the conversion efficiency and hinders its practical usage^9,10^. A breakthrough came that the optical damage resistance of LiNbO_3_ could be improved at least two orders of magnitude when 4.6 mol.% MgO was doped into^11,12^. From then on, MgO-doped LiNbO_3_ (Mg:LN) has become the often used material in frequency conversion, optical waveguide, optical parametric oscillation and terahertz source^13–17^. But for infrared-to-visible nonlinear frequency conversion, the phase-matching temperature is usually above 100 °C, so a precise temperature-controlled stove is needed. The extra unit in the laser cavity can increase the complexity of the system and lead to the additional energy loss^18^. Besides, the elevated operating temperature will also reduce components reliability and shorten service life. Detailed investigations show that the phase-matching temperature of Mg:LN increases with increased MgO concentration, and reaches the maximum near the doping threshold, then decreases with further increased doping concentration^19^. Therefore, it is possible that phase matching achieves at room temperature (about 25 °C) with a high enough doping concentration of MgO, but such heavily doped Mg:LN crystal is hard to grow with high optical quality^20^. Later, several ions such as Zn^2+^, In^3+^, Sc^3+^, and Hf^4+^ were also reported to have the same resistance against optical damage when doped into LiNbO_3_, but just like Mg:LN, the efficient noncritical phase matching at room temperature is still a problem^19,21–23^. Therefore, room temperature 90° phase-matching has always been a serious challenge for LiNbO_3_ at present.

In recent years, ZrO_2_-doped LiNbO_3_ (Zr:LN) has attracted great attentions because of its high optical damage resistance in the visible and even ultraviolet region, low doping threshold (2.0 mol.%) and distribution coefficient close to one^24–26^. Up to now, investigations on Zr:LN crystals mainly focus on optical waveguide, defect structure and co-doping with photorefractive impurities^27–29^, but their refractive indices and nonlinear optical properties (e.g., phase-matching temperature) are rarely reported. In fact, no sign of phase matching in Zr:LN crystals was found when they were heated from room temperature to above 200 °C in our pre-experiments, which implies that the phase-matching temperature of Zr:LN crystals may be lower than room temperature. If that is true, we may obtain room temperature 90° phase-matching by doubly doping with ZrO_2_ and MgO in LiNbO_3_. And co-doping with two optical damage resistant ions is conducive to finely tune the optical properties of LiNbO_3_^19,30^.

In this paper, we grew a series of ZrO_2_ and MgO co-doped LiNbO_3_ (Zr,Mg:LN) crystals with various ZrO_2_ dopants, and the doping concentration of MgO was chosen as 5.0 mol.% because Mg:LN has high phase-matching temperature in this doping level. Our experimental results demonstrate that the phase-matching temperature of Zr,Mg:LN monotonically decreases with increased ZrO_2_ concentration. And the efficient 90° phase-matching is achieved in Zr,Mg:LN crystal at room temperature without a stove, meanwhile it also has a very high optical damage resistance.

## Results

### Temperature tuned 90° phase-matching

**Figure 1:**
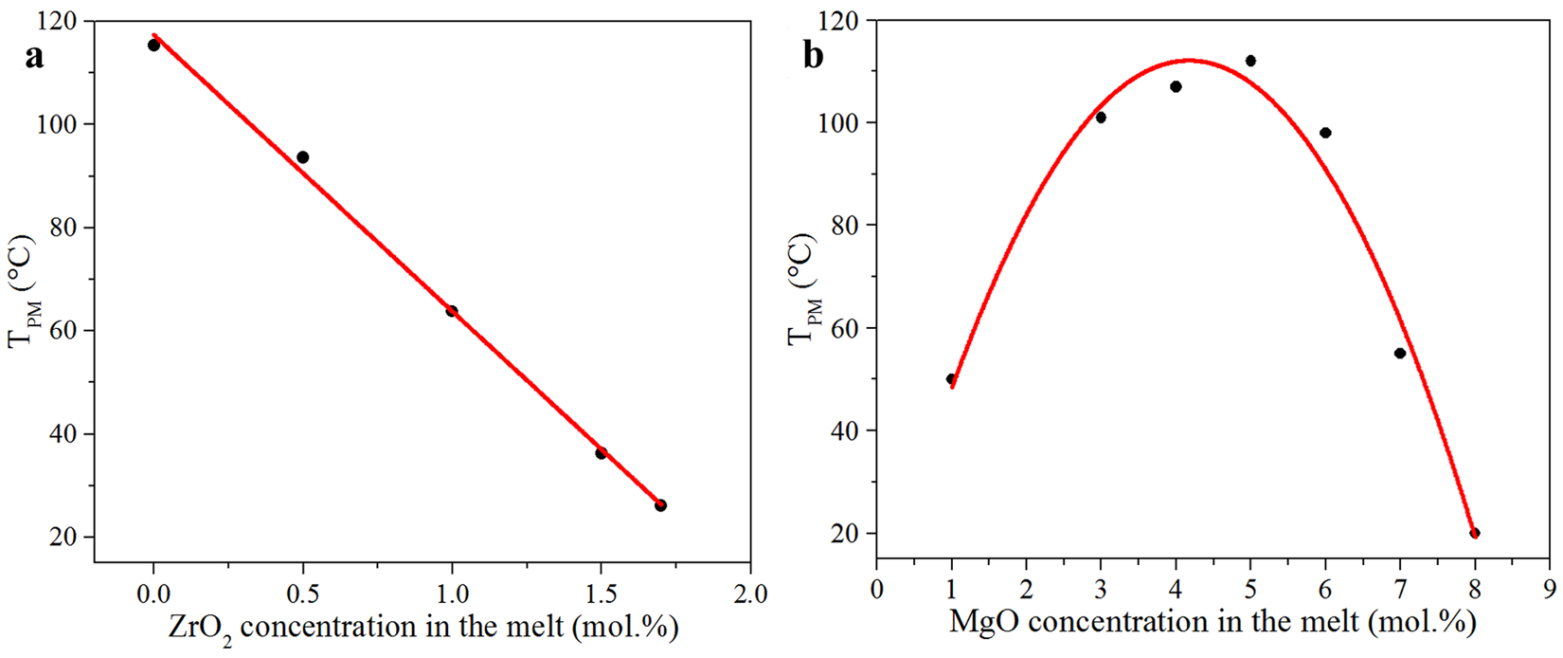
Phase-matching temperature *T*_PM_ versus the doping concentration in the melt of (**a**) Zr,Mg:LN and (**b**) Mg:LN crystals. The dotted symbols represent the experimental data, and the red guided line is a function fitting.

Temperature tuned 90° phase-matching was achieved by using a Q-switched Nd:YAG laser with a wavelength of 1064 nm. The dependence of the phase-matching temperature *T*_PM_ on the ZrO_2_ concentration in the melt for Zr,Mg:LN crystals is depicted in Fig. 1(a). From this figure, we can see that the *T*_PM_ decreases as the ZrO_2_ concentration increases, and a straight line can be fitted well to the experimental data. For comparison, the *T*_PM_ versus the doping concentration of MgO for Mg:LN crystals is presented in Fig. 1(b), and the data are referenced from the previous literature^19^. The relationship between the *T*_PM_ and the impurity concentration is similar to a parabola going downwards, and the maximum temperature stays within the concentration range of 4~6 mol.%. Generally, the *T*_PM_ versus the doping concentration exposes a more or less expressed threshold behavior corresponding to the sharp change of optical properties, and this similar behavior can be found in other mono or dual doped LN crystals, such as Zn:LN^21^, Sc:LN^22^ and Zn,In:LN^30^. In contrast, Zr,Mg:LN crystals exhibit a significant monotonic, and a simple linear extrapolation from existing data holds over a wider concentration range.

**Figure 2:**
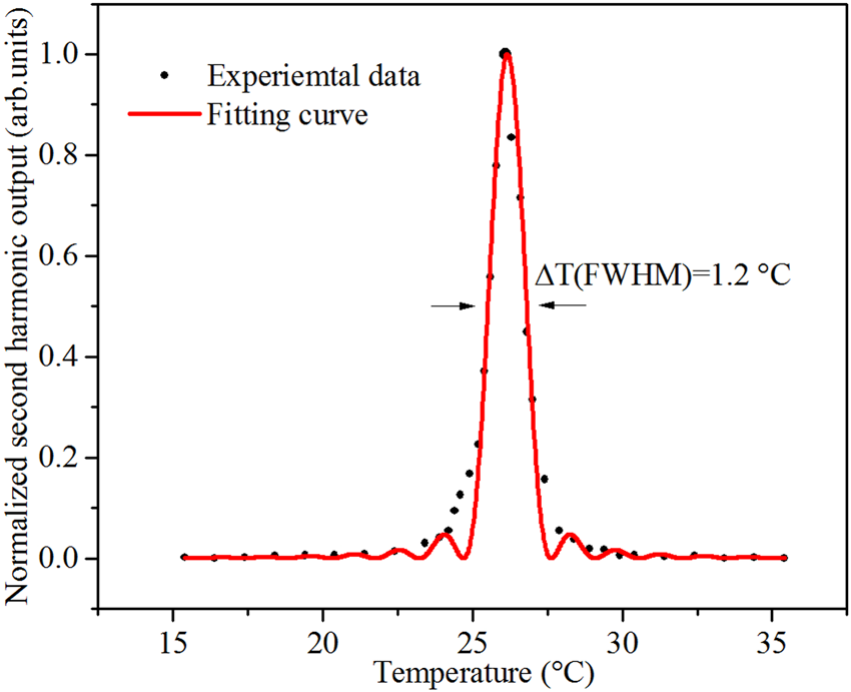
Normalized temperature-tuning curve for 90° phase-matching at 1064 nm in Zr_1.7_,Mg_5.0_:LN crystal. The dots are the measured second harmonic output at 532 nm, and the solid curve is fitted to $\,\mathrm{sinc}(x)={(\sin (x)/x)}^{2}$. The noncritical phase matching occurs at 26.1 °C with a FWHM of 1.2 °C.

**Figure 3:**
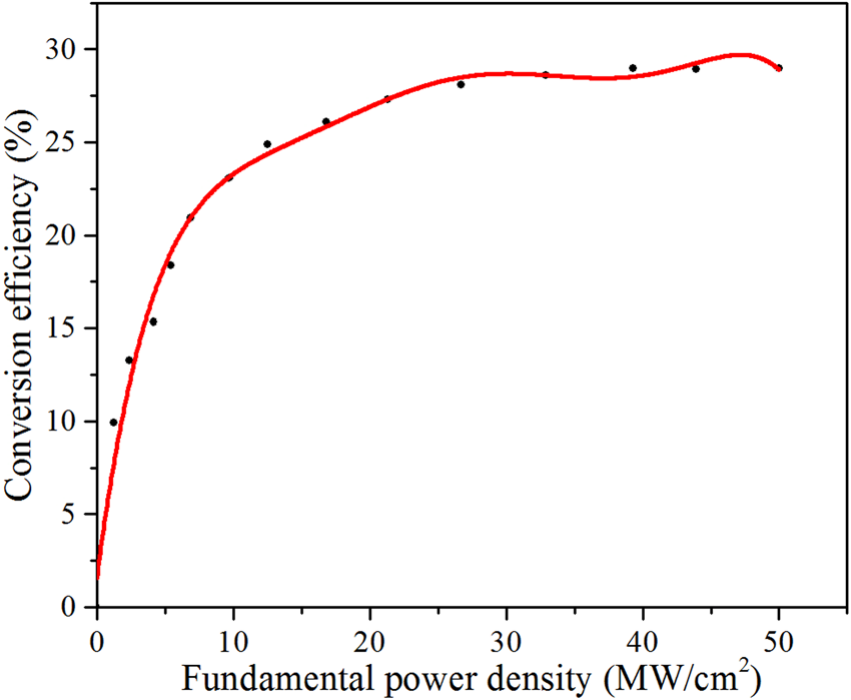
Power conversion efficiency of 90° phase-matched Zr_1.7_,Mg_5.0_:LN crystal as a function of the incident 1064 nm peak-power density. The solid curve is the guide to the eye.

Please note that the phase-matching temperature in Zr_1.7_,Mg_5.0_:LN crystal is 26.1 °C, which is close to room temperature (25 °C). Figure 2 clearly shows its normalized temperature-tuning curve for doubling 1064 nm using 90° phase-matching. The dots are the measured second harmonic output power, and the solid curve is a fit to the $\mathrm{sinc}(x)$ function, which almost perfectly overlaps the experimental data. The full width at half maximum (FWHM) of the temperature-tuning curve is 1.2 °C. Moreover, the conversion efficiency is plotted as a function of the incident fundamental power density in Fig. 3. An average second harmonic power of 91.5 mW is obtained with a conversion efficiency of 28.6% at the peak-power density of 50 MW/cm^2^, and maintaining this conversion efficiency for two hours, there is no significant degradation. Overall, we should point out emphatically that if considering the temperature increase (about 2~3 °C) in the continuous harmonic output^31,32^, Zr_1.7_,Mg_5.0_:LN crystal is particularly well-suited for practical application of laser frequency doubling at room temperature.

### Optical damage resistance

**Figure 4:**
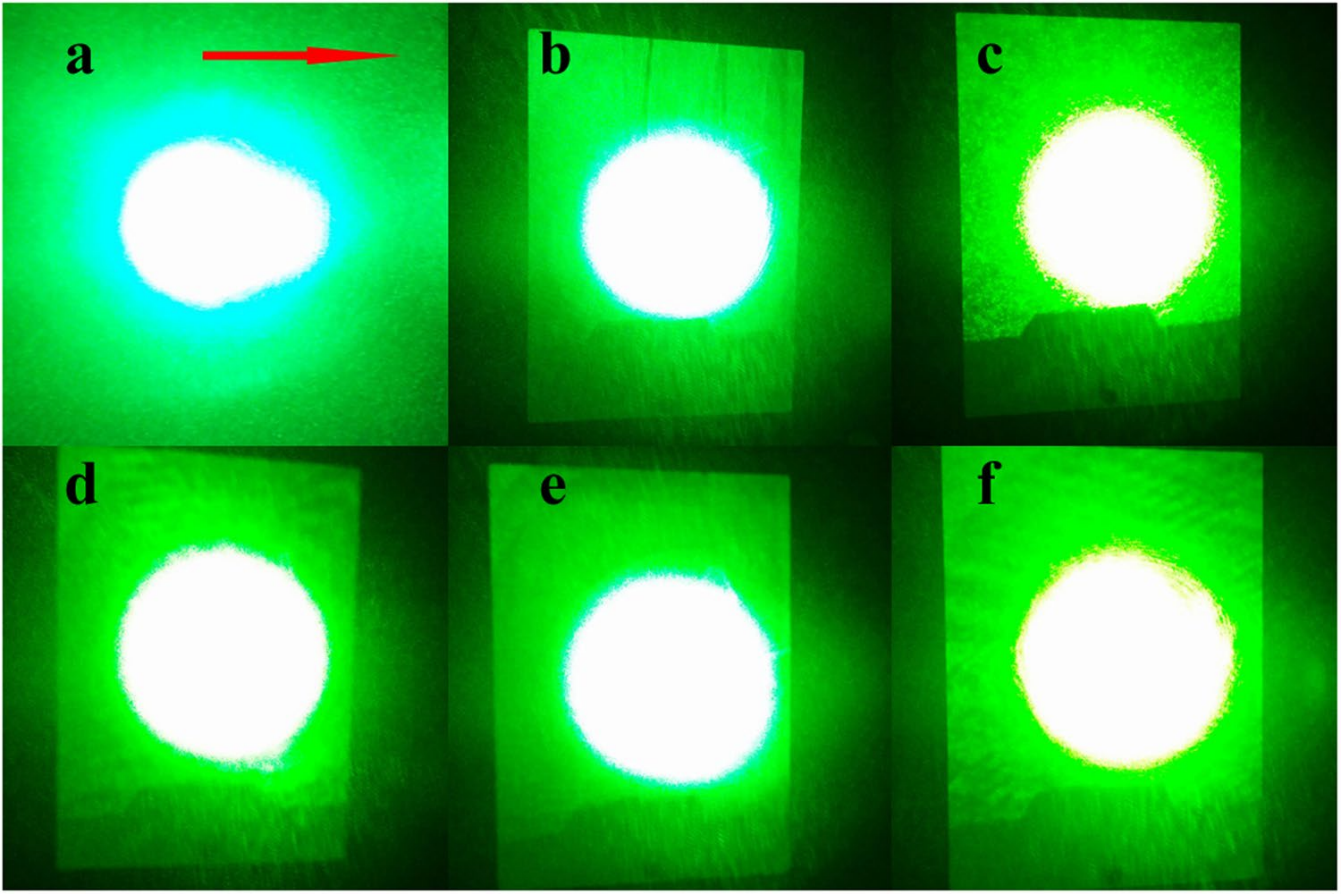
Transmitted laser beam spots after 5 min of irradiation. (**a**) Mg_5.0_:LN, (**b**) Zr_0.5_,Mg_5.0_:LN, (**c**) Zr_1.0_,Mg_5.0_:LN, (**d**) Zr_1.5_,Mg_5.0_:LN, (**e**) Zr_1.7_,Mg_5.0_:LN, (**f**) Zr_2.0_:LN. The light intensities are (**a**) 4.1 × 10^5^ W/cm^2^ and (**b**)–(**f**) 6.5 × 10^6^ W/cm^2^. The arrow direction represents the *c*-axis of the crystal.

In order to measure the optical damage resistance, the distortion of the transmitted light beam through the wafer was observed with a 532 nm laser. Figure 4 shows the transmitted laser beam spots after 5 min of irradiation. As the concentration of ZrO_2_ increases from 0.5 to 1.7 mol.%, none of Zr,Mg:LN crystals appears noticeable beam smeared, even under the highest focused intensity of 6.5 × 10^6^ W/cm^2^ in our laboratory, and the optical damage resistance is the same magnitude as that of Zr_2.0_:LN. However, Mg_5.0_:LN crystal can only withstand a maximum intensity of 4.1 × 10^5^ W/cm^2^ under the same conditions. The above results indicate that the optical damage resistance of these Zr,Mg:LN crystals is improved by at least an order of magnitude than that of Mg_5.0_:LN.

To quantitatively characterize the optical damage resistance, the light-induced changes of the refractive index Δ*n* of these crystals were measured by two-beam holography^33^. Two *e*-polarized coherent beams at 532 nm were intersected in the wafers with equal intensity of 400 mW/cm^2^. The change of refractive index Δ*n* was calculated by the equation^34^ ${\eta }_{{\rm{\max }}}={\sin }^{2}({\rm{\pi }}d{\rm{\Delta }}n/\lambda \,\cos \,{\theta }_{{\rm{cry}}})$. Here, ${\eta }_{{\rm{\max }}}$ is the maximum diffraction efficiency; $\lambda$ is the wavelength, 532 nm; *d* is the crystal thickness, 3.0 mm; and ${\theta }_{{\rm{cry}}}$ is the intersection half-angle of the two coherent beams outside the crystal, $2{\theta }_{{\rm{cry}}}$ = 30°. The photoconductivity ${\sigma }_{{\rm{ph}}}$ was also estimated through the relationship, ${\sigma }_{{\rm{ph}}}={\varepsilon }_{0}\varepsilon /{\tau }_{e}$, where ${\varepsilon }_{0}$ is the vacuum dielectric constant, *ε* = 28 is the relative dielectric constant of the crystal^35^, and the erasure time constant ${\tau }_{e}$ is defined as the time when the diffraction efficiency decays to 1/*e* of its initial value.

**Figure 5:**
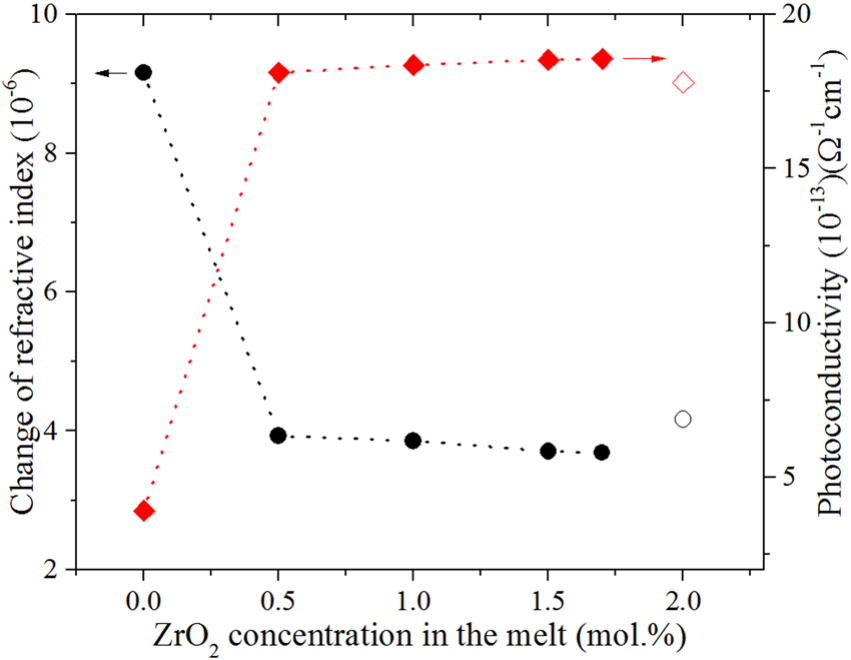
The change of refractive index Δ*n* and photoconductivity *σ*_ph_ as a function of the ZrO_2_ concentration in the melt for Zr,Mg:LN crystals. The open symbols represent the data of Zr_2.0_:LN for comparison.

The change of refractive index Δ*n* and the photoconductivity ${\sigma }_{{\rm{ph}}}$ versus the ZrO_2_ concentration for all samples are presented in Fig. 5. We can see that the change of refractive index reduces rapidly with doping 0.5 mol.% ZrO_2_ into Mg_5.0_:LN, then changes slightly as the ZrO_2_ concentration increases. Moreover, the Δ*n* of Zr,Mg:LN is considerably less than that of Mg_5.0_:LN and even lower than that of Zr_2.0_:LN. In addition, the ${\sigma }_{{\rm{ph}}}$ of Zr,Mg:LN is larger than that of Mg_5.0_:LN but close to that of Zr_2.0_:LN. It is well known that the increase of the photoconductivity is primarily responsible for the increase of the optical damage resistance^36^. Therefore, the results demonstrate again that adding some ZrO_2_ into Mg_5.0_:LN can further enhance the optical damage resistance, which is consistent with the results of the transmitted light beam distortion.

### Infrared absorption spectra

**Figure 6:**
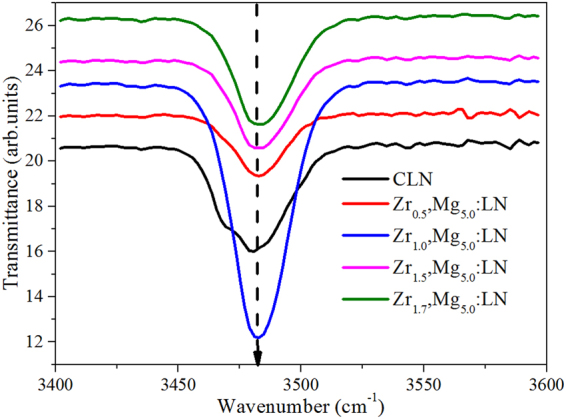
The OH^−^ absorption spectra of Zr,Mg:LN crystals. For comparison, that of CLN is also drawn. The dashed arrow marks the position of absorption peaks.

The infrared absorption spectra, referring mainly to OH^−^ absorption spectra, sensitively reflect the change of defect structure in LiNbO_3_, which have become an important tool in studying the properties of dopant-related defects. Figure 6 shows the OH^−^ absorption spectra of CLN and Zr,Mg:LN crystals. As no obvious OH^−^ band shift is observed, a three-peak model^37^ is employed by Lorentz fitting, and the results are listed in Table 1. For comparison, that of Mg_5.0_:LN and Zr_2.0_:LN crystals are also listed. It can be seen from the table that the component peaks of Zr_2.0_:LN are located at 3475, 3485, and 3495 cm^−1^, respectively, which agree with the previous results^38^, and the shift in component peaks is connected with the formation of OH^−^–${{\rm{Zr}}}_{{\rm{Nb}}}^{-}$ clusters. Moreover, with increased doping concentration of ZrO_2_, the relative intensity of OH^−^–${{\rm{Zr}}}_{{\rm{Nb}}}^{-}$ band will increase^39^. As shown in the table, the peak of 3476 cm^−1^ appears in Zr_0.5_,Mg:LN and moves to 3478 cm^−1^ with increased concentration of ZrO_2_. Based on these analyses, the total content of 0.5 mol.% ZrO_2_ and 5.0 mol.% MgO in LiNbO_3_ has reached the doping threshold.

**Table 1:** The position of component peaks of the OH^−^ absorption spectra.

Sample	Position of peaks (cm^−1^)		
CLN	3468	3481	3490
Mg_5.0_:LN		3535	
Zr_2.0_:LN	3475	3485	3495
Zr_0.5_,Mg_5.0_:LN	3476	3484	3493
Zr_1.0_,Mg_5.0_:LN	3477	3485	3493
Zr_1.5_,Mg_5.0_:LN	3478	3486	3495
Zr_1.7_,Mg_5.0_:LN	3478	3487	3495

### UV-visible absorption spectra

**Figure 7:**
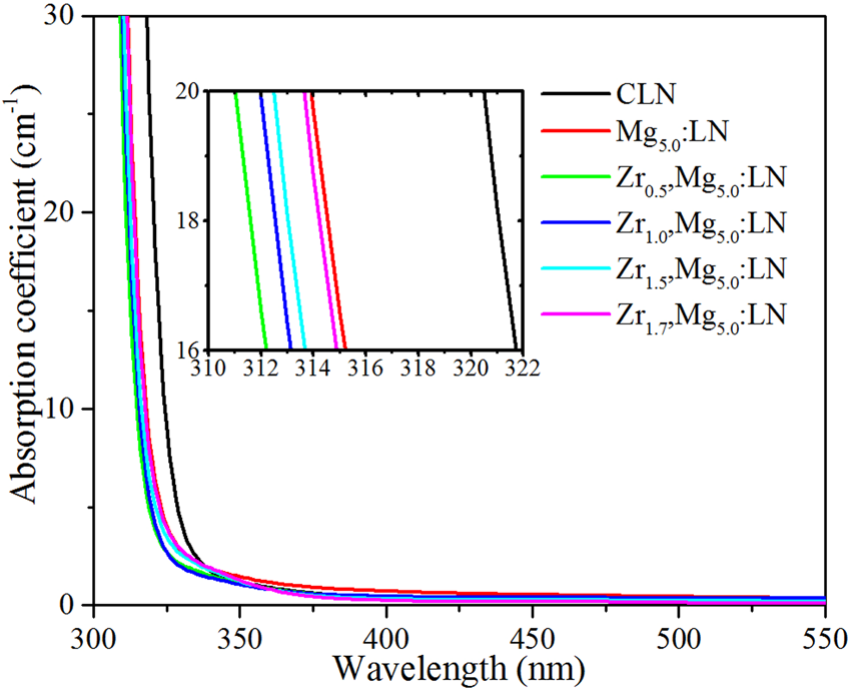
The UV-visible absorption spectra of Zr,Mg:LN crystals and of CLN and Mg_5.0_:LN for comparison.

The UV absorption edge of LiNbO_3_ is sensitive to the crystal composition and defect^40^, especially the absorption edge has a maximum violet-shift when the doping concentration reaches the threshold. The UV-visible absorption spectra of Zr,Mg:LN crystals are shown in Fig. 7. The inset clearly shows the position of absorption edges. Here, the absorption edge is defined as the wavelength where the absorption coefficient is equal to 20 cm^−1^. From this figure, we can see that the absorption edge of Zr_0.5_,Mg_5.0_:LN crystal has a maximum violet-shift, and with the doping concentration of ZrO_2_ increasing from 0.5 to 1.7 mol.%, the absorption edge has an obvious red-shift. Therefore, we can conclude that the total doping concentration of 0.5 mol.% ZrO_2_ and 5.0 mol.% MgO in LiNbO_3_ reaches the threshold level, which is in agreement with the results of OH^−^ absorption spectra.

## Discussion

The absorption spectra of Zr,Mg:LN crystals demonstrate that the total content of 0.5 mol.% ZrO_2_ and 5.0 mol.% MgO has reached the doping threshold, which implies that impurity ions enter normal Nb sites after all ${{\rm{Nb}}}_{{\rm{Li}}}^{4+}$ ions have been substituted. Moreover, the three-peak fitting analysis of OH^−^ absorption spectra indicates that the peak shifts are related to the formation of OH^−^–${{\rm{Zr}}}_{{\rm{Nb}}}^{-}$ complexes, and 3535 cm^−1^ absorption peak^36^ attributed to the OH^−^ stretching vibration of ${{\rm{Mg}}}_{{\rm{Li}}}^{+}$–OH^−^–${{\rm{Mg}}}_{{\rm{Nb}}}^{{\rm{3}}-}$ complex is not observed. These results indicate that the additional ZrO_2_ can directly influence the threshold level of MgO, namely, Zr^4+^ ions affect the site occupation of Mg^2+^ ions. So it can be deuced that the appearance of ${{\rm{Mg}}}_{{\rm{Li}}}^{+}$ + ${{\rm{Zr}}}_{{\rm{Nb}}}^{-}$ neutral pairs leads to no obvious OH^−^ absorption band shift. And the amount of ${{\rm{Mg}}}_{{\rm{Li}}}^{+}$ + ${{\rm{Zr}}}_{{\rm{Nb}}}^{-}$ complexes will increase with increased concentration of ZrO_2_. Considering the ${{\rm{Mg}}}_{{\rm{Li}}}^{+}$ + ${{\rm{Zr}}}_{{\rm{Nb}}}^{-}$ pairs without charge compensation, electron trap centers will dramatically reduce in crystals, which brings about the great increase of photoconductivity then results in strong optical damage resistance.

Our results on SHG of Nd:YAG lasers have shown that the additional ZrO_2_ doping can significantly influence the phase-matching temperature of Mg_5.0_:LN. In particular, the monotonic decrease of phase-matching temperature on ZrO_2_ concentration is found for the first time, which is different from the previous reports on doped LiNbO_3_. As we know, so-called optical damage resistant impurities in visible region such as Mg, Zn, In and Hf can enhance the UV photorefractive effect^41,42^, but Zr exhibits excellent optical damage resistance in both visible and UV region. It is thought that the enhanced UV photorefractive effect has direct relationship with doped ions occupying Nb sites^43^, which means that Zr^4+^ ions in Nb sites can greatly alter defect structures and properties of LiNbO_3_. In Zr,Mg:LN crystals, ${{\rm{Mg}}}_{{\rm{Li}}}^{+}$ + ${{\rm{Zr}}}_{{\rm{Nb}}}^{-}$ neutral pairs may play an important role in this monotonic decrease relationship. However, further investigation is greatly needed to clarify the micro-mechanism.

## Conclusion

We grew a series of LiNbO_3_ co-doping with 5.0 mol.% MgO and various ZrO_2_ concentrations. The experimental results indicate that the phase-matching temperature of Zr,Mg:LN decreases with increased ZrO_2_ concentration for the first time. And 90° phase-matching of 1064 nm radiation is achieved at room temperature in Zr_1.7_,Mg_5.0_:LN crystal, while it holds a high resistance of optical damage at 532 nm, and does not suffer any dark trace damage when exposed to high power laser irradiation for two hours, which will be greatly valuable for engineering applications in compact and efficient high-power green lasers. These excellent properties of Zr,Mg:LN may be attributed to the formation of ${{\rm{Mg}}}_{{\rm{Li}}}^{+}$ + ${{\rm{Zr}}}_{{\rm{Nb}}}^{-}$ neutral defect pairs.

## Methods

### Samples preparation

A series of doubly doped LiNbO_3_ crystals were grown along the *c*-axis by the conventional Czochralski method in air. 0, 0.5, 1.0, 1.5 and 1.7 mol.% ZrO_2_ were added, respectively, to the congruent melt (Li/Nb = 48.38/51.62) doping with 5.0 mol.% MgO, labeled as Mg_5.0_:LN, Zr_0.5_,Mg_5.0_:LN, Zr_1.0_,Mg_5.0_:LN, Zr_1.5_,Mg_5.0_:LN and Zr_1.7_,Mg_5.0_:LN. After annealing treatment and artificial polarization, the crystals were cut into 3.0 mm and 1.0 mm thick *y*-plates for the characterization of photorefraction and absorption. Choosing crack-free and uniform bulk crystals were cut into 9.0 × 10.0 × 9.0 mm^3^ (X × Y × Z) for second harmonic generation, where *y*-axis was the transmission direction. All samples were optical grade polished on both faces perpendicular to *y*-axis of the crystal. For comparison, congruent LiNbO_3_ and 2.0 mol.% ZrO_2_-doped congruent LiNbO_3_ crystals were also prepared, labelled as CLN and Zr_2.0_:LN, respectively.

### Spectra characterization

The infrared absorption spectra and UV-Visible absorption spectra of 1.0 mm thick *y*-plates were measured at room temperature with a Magna-560 Fourier transform infrared spectrophotometer and a U-4100 spectrophotometer, respectively. The resolution value of this infrared spectrometer was 4.0 cm^−1^, and the step-length of the UV-Vis. spectrometer was 1.0 nm.

### Second harmonic generation

The experiments for second harmonic generation were performed with a Q-switched Nd:YAG laser 1064 nm at a 1 Hz repetition rate, 8 ns pulse width. The laser facula diameter was 5.0 mm, and the maximum average output energy was 320 mJ. The fundamental light was directed to the crystal in a geometry with the *c*-axis of the crystal perpendicular to the polarization direction of the light, so-called 90° phase-matching. The second harmonic energy was detected with a band-pass filter and a pulse laser energy meter. The bulk crystal was mounted in an oven thermally controlled to a temperature better than ±0.2 °C. The temperature was measured with a Pt-100 thermistor placed in direct contact with the crystal, and a slow heating rate was used about 0.5 °C/min to minimize temperature gradients with the sample. In addition, the distance between the entrance and the exit windows of the crystal surfaces was at least 5.0 cm to minimize the possible temperature gradients along the crystal *y*-axis. The formula for the conversion efficiency was *η* = *E*_2_/*E*_1_, where *E*_1_ was the energy of the fundamental wave and *E*_2_ was that of the second harmonic wave.

### Data availability

The datasets generated during the current study are available from the corresponding author on reasonable request.
